# Comment on: “Inverse relationship between serum haptoglobin and acute kidney injury in critically ill patients with sepsis: A retrospective cohort study of the MIMIC-IV 3.0 database”

**DOI:** 10.1016/j.clinsp.2025.100781

**Published:** 2025-09-22

**Authors:** Songsong Luo, Xiaoyuan Shen

**Affiliations:** Department of Critical Care Medicine, The First People’s Hospital of Xiaoshan District, Xiaoshan Affiliated Hospital of Wenzhou Medical University, Hangzhou, Zhejiang, China

Dear Editor,

We read with great interest the recent study by Liao et al. (2025),^1^ which examined the association between serum haptoglobin levels and Acute Kidney Injury (AKI) among critically ill patients with sepsis, utilizing the MIMIC-IV database. The authors addressed an important clinical question and applied high-resolution electronic health data to evaluate a potential early biomarker for Sepsis-Associated AKI (SA-AKI). While the findings provide meaningful insights, several methodological aspects and translational considerations warrant further discussion.

First, although the retrospective analysis based on MIMIC-IV offers strong granularity, its single-center origin derived from a U.S. academic hospital may limit the generalizability of the conclusions. Patient populations in other health systems may differ in racial composition, genetic background, and environmental exposures, all of which can influence haptoglobin expression. Thus, external validation in multicenter and multiethnic cohorts is essential before considering clinical application.

Second, the hypothesis that haptoglobin mitigates AKI by scavenging free hemoglobin is biologically plausible. However, the absence of plasma-free hemoglobin data in MIMIC-IV restricts direct evaluation of this proposed mechanism. Since haptoglobin’s primary function is to neutralize the oxidative effects of circulating free hemoglobin, future studies should incorporate markers of hemolysis and oxidative stress to reinforce biological plausibility and improve causal inference.

Third, the use of early haptoglobin measurements aligns well with the clinical goal of timely AKI risk stratification. However, several critical clinical variables known to influence AKI development were not included in the analysis. These include fluid balance status, vasopressor requirements, nephrotoxic drug exposure (e.g., vancomycin, iodinated contrast media), and dynamic hemodynamic instability.^2^^,^^3^ The omission of these factors may introduce residual confounding and limit the applicability of the findings to real-world ICU settings. Additionally, the use of the SOFA score as a covariate warrants caution, as it includes serum creatinine ‒ a key component of AKI diagnosis. Including SOFA in models that predict AKI may result in statistical overadjustment and obscure the true relationship between haptoglobin and AKI. A modified SOFA score excluding the renal component could offer a more accurate adjustment for illness severity while avoiding collinearity with the outcome.

Although the study presents haptoglobin as a promising early biomarker for sepsis-associated AKI, its clinical application remains limited due to factors such as short half-life, hepatic influence, and limited use in ICUs. A single baseline value may not reflect dynamic changes, and serial measurements alongside other biomarkers may be needed. Overall, the study offers valuable preliminary evidence, but further research is necessary to validate its findings, address confounders, and clarify underlying mechanisms to support its use in critical care.

## Ethical approval

Not applicable.

## Declaration of generative AI and AI-assisted technologies in the writing process

During the preparation of this manuscript, the author used ChatGPT to assist with language refinement and stylistic editing. All AI-assisted content was carefully reviewed and revised by the author, who assumes full responsibility for the final version of the manuscript.

## Funding

No funding was received for this research.

## CRediT authorship contribution statement

**Songsong Luo:** Conceptualization, Writing – original draft. **Xiaoyuan Shen:** Supervision, Writing – review & editing.

## Declaration of competing interest

The authors declare no conflicts of interest.
